# Changes in cortical plasticity induced by paired associative stimulation in people with mild cognitive impairment and Alzheimer’s disease: a systematic review and meta-analysis

**DOI:** 10.3389/fncir.2026.1853684

**Published:** 2026-06-29

**Authors:** Xiaying Fu, Xiaomin Niu, Xiaoying Lin, Wenqi He, Chunyang Liao, Tiantian Xin, Shuqin Li, Sharie Xiao Wang, Jae Q. J. Liu, Bin Li, Jianghua Cheng

**Affiliations:** 1 Department of Rehabilitation Medicine, South China Hospital, Medical School, Shenzhen University, Shenzhen, Guangdong, China; 2 School of Rehabilitation Medicine, Gannan Medical University, Ganzhou, Jiangxi, China; 3 Department of Rehabilitation Sciences, The Hong Kong Polytechnic University, Hung Hom, Hong Kong SAR, China; 4 Department of Medical Administration, South China Hospital, Medical School, Shenzhen University, Shenzhen, Guangdong, China

**Keywords:** Alzheimer’s disease, cortical plasticity, mild cognitive impairment, motor-evoked potential, paired associative stimulation

## Abstract

**Background:**

Paired associative stimulation (PAS) is a non-invasive neuromodulation paradigm capable of inducing long-term potentiation (LTP)- or long-term depression (LTD)-like plasticity. It has been used to probe neuroplasticity and corticospinal excitability alterations in Alzheimer’s disease (AD) and mild cognitive impairment (MCI). However, existing studies report inconsistent directions and magnitudes of PAS-induced plasticity changes across the AD continuum.

**Methods:**

We searched PubMed, Web of Science, Embase, and the Cochrane Library from database inception to December 2025. Eligible studies were case–control studies and randomized controlled trials (RCTs) that assessed PAS-induced cortical plasticity in individuals with AD or MCI, with healthy older adults as controls. Study quality was evaluated using the Newcastle–Ottawa Scale (NOS) for non-randomized studies and the Cochrane Risk of Bias tool (RoB 2) for RCTs. This review was registered in PROSPERO (CRD420251178441).

**Results:**

Five studies met the inclusion criteria. Four studies quantified motor cortical plasticity using changes in motor-evoked potential (MEP) amplitude (total *N* = 135; 64 MCI/AD and 71 controls). The pooled analysis showed no significant difference in PAS-induced MEP changes between participants with MCI/AD and healthy controls [mean difference (MD) = 0.06, 95% CI (−0.05, 0.17), *p* = 0.32]. This pooled estimate was restricted to MEP-based outcomes and should be interpreted as an exploratory synthesis of motor-system readouts rather than definitive evidence of preserved cortical plasticity in MCI or AD. The remaining RCT, which assessed dorsolateral prefrontal cortex (DLPFC) plasticity using a repetitive PAS (rPAS) intervention without MEP outcomes, also found no significant improvement in prefrontal plasticity relative to control stimulation.

**Conclusion:**

Based on the currently available MEP-based evidence, PAS-induced motor-system plasticity findings in individuals with MCI or AD remain inconclusive. Given the limited number of studies and heterogeneity in experimental designs, the pooled negative results should not be interpreted as evidence that cortical plasticity is preserved. Corticospinal hyperexcitability and network-level PAS findings should be regarded as preliminary, hypothesis-generating observations requiring validation in larger, longitudinal, and biomarker-characterized cohorts.

## 1 Introduction

Alzheimer’s disease (AD) is the most common neurodegenerative cause of dementia, characterized by progressive cognitive decline and loss of daily functioning, posing a heavy burden on global healthcare systems (Jack et al., 2018). Amnestic mild cognitive impairment (aMCI) is the most common type of mild cognitive impairment (MCI), considered as a preclinical precursor of AD, and serves as a critical intervention window to alter the disease progression (Petersen et al., 2018). One of the core mechanisms underlying cognitive decline in AD is considered to be a dysfunction in synaptic plasticity, which refers to the brain’s ability to dynamically adjust synaptic connection strengths based on experience (Selkoe, 2002; Terry et al., 1991). At the cellular level, a typical model of this plasticity is long-term potentiation (LTP) in the hippocampus and neocortex, in which synaptic strength is persistently enhanced and is considered a fundamental physiological substrate for learning and memory formation (Bliss and Collingridge, 6407). Numerous studies have confirmed that AD-related pathological changes, such as amyloid-*β* (A*β*) oligomers and tau protein, can directly impair the induction and maintenance of LTP while enhancing long-term depression (LTD), thereby disrupting the cellular mechanisms of memory consolidation (Shankar et al., 2008; Hoover et al., 2010).

*In vivo* assessment of LTP in the human brain has long been challenging. Paired associative stimulation (PAS) is an non-invasive brain stimulation paradigm that temporally pairs peripheral nerve electrical stimulation with transcranial magnetic stimulation (TMS) of the cortex (Stefan et al., 2000). According to Hebbian theory, this precise timing can induce synaptic plasticity changes similar to LTP or LTD, and the degree of these changes is quantified by measuring the amplitude changes of motor evoked potentials (MEPs) (Wolters et al., 2003; Ziemann et al., 2006). More recently, the scope of PAS has expanded considerably beyond this classical sensorimotor paradigm. As comprehensively reviewed by Guidali et al. (2021a), novel “modified” PAS protocols have been developed, which can be categorized according to the nature of the paired stimuli: within-system PAS, cross-system PAS, and cortico-cortical PAS. These protocols have enabled the investigation of timing-dependent plasticity not only in sensorimotor networks but also in higher-order cognitive networks, including frontal cortices subserving memory, attention, and decision-making (Guidali et al., 2021b). This methodological expansion has positioned PAS as a versatile tool for probing circuit-specific plasticity across different brain regions in both healthy and pathological conditions. The PAS effect relies on the activation of postsynaptic N-methyl-D-aspartate (NMDA) receptors and shares the same neurophysiological mechanisms with other stimulation paradigms such as theta burst stimulation (TBS) (Huang et al., 2005; Di Lazzaro et al., 2008; Suppa et al., 2016). Thus, PAS provides a unique non-invasive observation window *in vivo* for people with MCI or AD to study cortical synaptic plasticity function.

In recent years, PAS has been employed in a number of studies to explore neuroplasticity in AD and MCI patients, but the findings have been inconsistent (Terranova et al., 2013; Lahr et al., 2016; Meder et al., 2021; Di Lorenzo et al., 2018; Kumar et al., 2023). Most studies have reported that PAS-induced LTP-like plasticity is significantly reduced or even converted to LTD-like inhibition in AD and MCI patients compared to healthy older adults (Battaglia et al., 2007; Koch et al., 2012), supporting the hypothesis of a “plasticity deficit” in AD. However, some investigations have reported contrary or more complex results. For example, some AD patients have exhibited excessive plasticity responses, possibly reflecting compensatory mechanisms or intrinsic heterogeneity across disease stages or subtypes (Motta et al., 2018). This heterogeneity may be modulated by factors such as individual cognitive reserve, age-related decline in cortical structural integrity (List et al., 2013), and the regulation of stimulus–response metaplasticity by the brain’s intrinsic state (Müller-Dahlhaus and Ziemann, 2015). In addition, widespread dysfunction of gamma-aminobutyric acid-ergic inhibitory interneurons in AD has also been proposed as a key contributor to abnormal plasticity responses (McDonnell et al., 2006; Miyaguchi et al., 2017). These conflicting findings may stem from differences in study design, stimulation parameters (e.g., PAS intervals), targeted brain regions, patient characteristics, and assessment methods.

To date, there has been no systematic integration or review of the existing evidence. Clarifying the patterns of PAS-induced cortical plasticity changes across people with MCI or AD has theoretical importance for understanding AD pathophysiology. However, given the limited evidence base, PAS-related measures should be considered candidate neurophysiological indicators rather than established diagnostic or prognostic biomarkers, consistent with broader cautions regarding the clinical translation of non-invasive brain stimulation measures in AD (Freitas et al., 2011a). Therefore, this systematic review and meta-analysis was conducted to synthesize the available evidence, clarify sources of heterogeneity, and cautiously evaluate whether PAS-induced MEP changes or related neurophysiological measures differ between people with MCI/AD and healthy controls.

## 2 Methods

### 2.1 Statement

This systematic review and meta-analysis followed the PRISMA 2020 guidelines and the methodological procedures recommended by the Cochrane Handbook (Page et al., 2020). Two reviewers (Xiaying Fu and Xiaoying Lin) independently performed literature screening, data extraction, quality assessment, and statistical analysis. Any disagreements were resolved through discussion with a third reviewer (Jianghua Cheng). Registration number: CRD420251178441.

### 2.2 Search strategy

We performed systematic literature searches in PubMed, Web of Science, Embase, and the Cochrane Library up to December 2025. Using the following search terms in combination: (paired associative stimulation OR PAS OR cortico-peripheral stimulation) AND (Alzheimer disease OR Alzheimer’s disease OR AD OR dementia OR mild cognitive impairment OR cognitive impairment).

### 2.3 Inclusion criteria

Two reviewers (Xiaying Fu and Xiaoying Lin) independently selected studies and decided on inclusion; disagreements were resolved by a third reviewer (Jianghua Cheng). We included studies that met all of the following criteria: (1) Participants diagnosed with Alzheimer’s disease or MCI by any clinical criteria, e.g., National Institute of Neurological and Communicative Disorders and Stroke and the Alzheimer’s Disease and Related Disorders Association (NINCDS-ADRDA), Diagnostic and Statistical Manual of Mental Disorders (DSM) or the National Institute on Aging-Alzheimer’s Association (NIA-AA) 2011 criteria, with a control group or sufficient pre-post intervention data; (2) Use of PAS paradigms including conventional PAS, cortico-cortical PAS(cc-PAS) or rapid PAS to measure cortical plasticity, or studies that used PAS as an intervention were also included if they reported electrophysiological outcome measures (e.g., MEP changes) to assess plasticity. Studies had to report quantitative data on measures such as motor threshold, intracortical inhibition, afferent inhibition, cortical silent period (CSP), motor evoked potential (MEP) amplitudes, intracortical facilitation (ICF), and/or central motor conduction time; (3) Comparison of cortical plasticity between MCI and/or AD patients and older adults; (4) Studies reporting quantitative or clearly extractable neurophysiological or cognitive outcomes related to PAS, including MEP amplitude, motor threshold, short-latency afferent inhibition (SAI), intracortical facilitation (ICF), cortical silent period (CSP), dorsolateral prefrontal cortex (DLPFC) plasticity, oscillatory coupling, or cognitive task performance; (5) Study design of case–control, cohort, or randomized controlled trial; (6) Published in English.

### 2.4 Exclusion criteria

If the study does not meet all the inclusion criteria, it will be excluded. The specific criteria include: (1) Studies in which AD or MCI participants were absent, or in which AD/MCI data could not be separated from other neurological or psychiatric populations. Studies including additional patient groups were not excluded when extractable AD/MCI data and appropriate control comparisons were available; (2) animal or *in vitro* studies; (3) Studies that did not employ a PAS paradigm to assess or modulate cortical plasticity; (4) Studies lacking a healthy older adult control group or interventional studies without a parallel control group; (5) Studies that failed to report any extractable neurophysiological or cognitive outcome related to PAS; (6) Non-original research designs such as reviews, meta-analyses, study protocols, or conference abstracts; and articles for which a full-text manuscript in English was not available.

### 2.5 Data extraction

Three authors (Xiaying Fu, Xiaoying Lin, and Jianghua Cheng) independently extracted data; disagreements were resolved through discussion. Extracted data included sample sizes, sample characteristics (e.g., age, sex, diagnosis); PAS protocols (e.g., target area, stimulation parameters); statistical data evaluating plasticity (e.g., effect sizes, *p*-values); and outcome changes (e.g., MEP amplitude change, cognitive score change). When published data were insufficient, original study authors were contacted for additional information.

### 2.6 Risk of bias assessment in individual studies

Since included studies comprised case–control trials and a randomized controlled trial, we assessed bias risk using different tools for each design. For the four case–control studies, we used the Newcastle-Ottawa Scale (NOS) to evaluate risk of bias in non-randomized studies. Specifically, we scored studies on selection of study groups (up to 4 points), comparability of groups (up to 2 points), and ascertainment of exposure (up to 3 points). Higher scores indicate better quality: we considered NOS scores of 8–9 to indicate high-quality studies, 6–7 good quality, 5 moderate quality, and ≤ 4 low quality. The risk of bias for the single included RCT was assessed with the Cochrane Risk of Bias 2 (RoB 2) tool. This tool covers five domains: the randomization process, deviations from intended interventions, missing outcome data, outcome measurement, and selection of reported results.

### 2.7 Statistical analysis

Because PAS protocols differed substantially in stimulation target, pairing method, stimulation frequency, and outcome definition, quantitative pooling was restricted to studies reporting extractable MEP amplitude changes after PAS in patient and healthy control groups. The prefrontal rPAS randomized trial, which did not report comparable MEP-based outcomes, was summarized narratively. For continuous outcomes measured on the same scale, mean difference (MD) and 95% confidence intervals (CIs) were calculated. Pooled effect sizes were estimated using a random-effects model to account for between-study heterogeneity. Heterogeneity was assessed with the *I^2^* statistic and Chi-squared test. Given the small number of studies, formal subgroup analyses and publication-bias tests were not considered reliable. All analyses were performed in RevMan 5.3. The pooled estimate was interpreted as an exploratory synthesis of MEP-based motor-system readouts rather than as a definitive measure of global cortical plasticity.

## 3 Results

### 3.1 Search results

**Figure 1:**
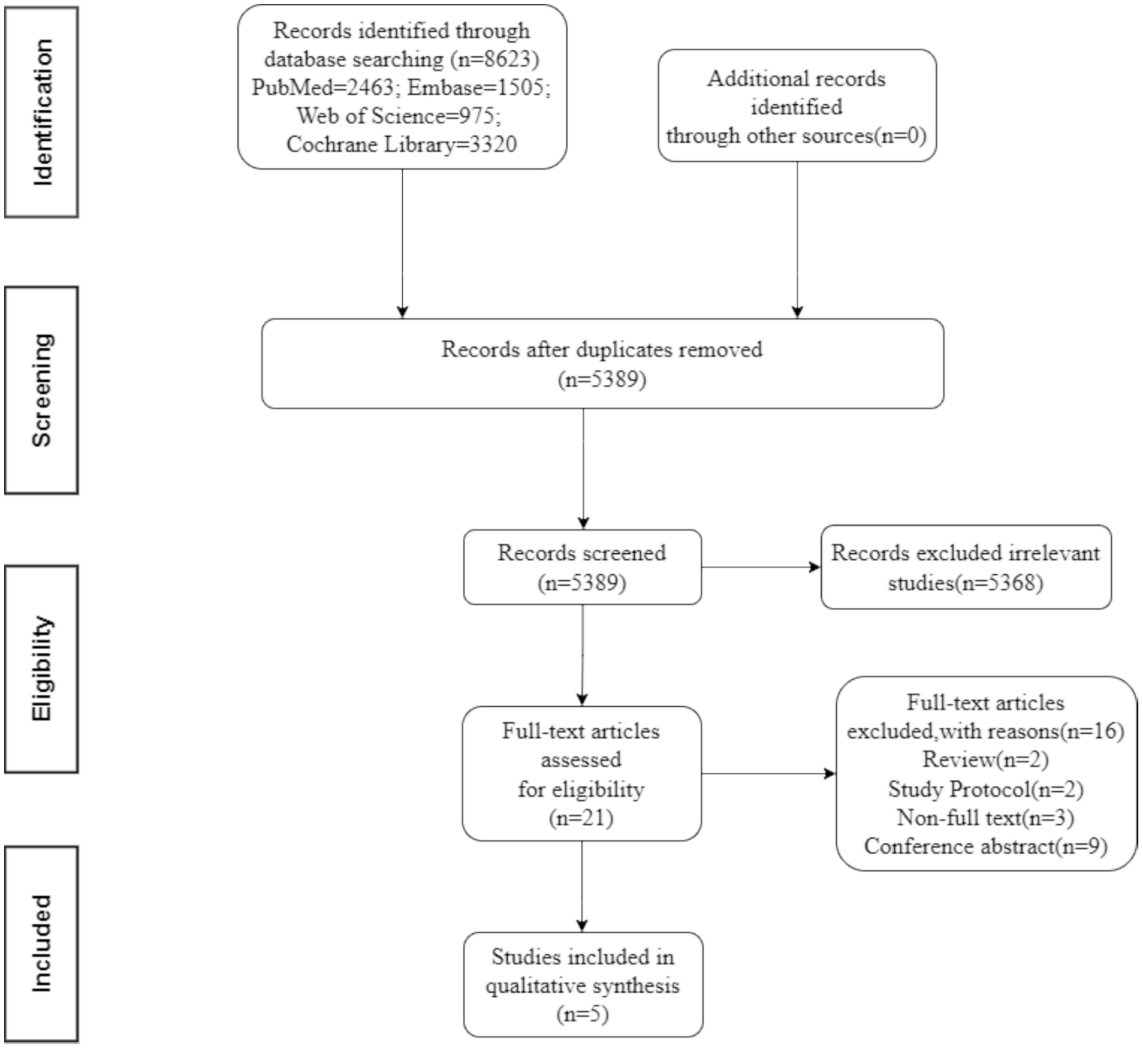
Flow diagram showing the screening process and the search results.

The database search initially identified 8,263 published records, of which 21 were selected for full-text review after screening titles and abstracts. Among these, 16 studies were excluded (2 reviews, 2 study protocols, 9 conference abstracts, 3 with no full text available). The remaining 5 studies met the inclusion criteria and were included in this meta-analysis (see Figure 1).

### 3.2 Study characteristics

The five included studies (Terranova et al., 2013; Lahr et al., 2016; Meder et al., 2021; Di Lorenzo et al., 2018; Kumar et al., 2023) comprised 182 participants (mean age 72.4 years; ~ 54% female). Of these, 56 were AD patients, 39 were MCI patients, and 87 were cognitively normal older adults. Key characteristics of the included studies are summarized in Tables 1–3. The included studies were published between 2013 and 2023. Among these, one study employed a high-frequency, short-duration 5 Hz rapid PAS (5 Hz-rPAS) intervention, delivering 600 paired stimuli over 2 min; this protocol’s shorter duration may impose a lower demand on participants’ sustained attention compared to traditional paradigms. In contrast, the other four studies used traditional low-frequency PAS paradigms, with stimulation frequencies of 0.1–0.25 Hz over 20–30 min; such protocols are known to require greater attentional engagement. In terms of study design, one study was a RCT, while the remainder were case–control studies. Regarding target populations, 1 study only included patients with MCI, 3 only included patients with AD, and 1 included both AD and MCI patients. As for stimulation targets, one study targeted the prefrontal cortex with a focus on higher cognitive functions, whereas the other four targeted the motor cortex or its connected networks, focusing on sensorimotor integration or basic plasticity mechanisms.

**Table 1:** Characteristics of the population in the included studies.

Characteristics	Terranova et al. (2013)	Kumar et al. (2023)	Di Lorenzo et al. (2018)	Lahr et al. (2016)	Meder et al. (2021)
Country	Italy	Canada	Italy	Germany	Germany
Study design	Case control	RCT	Case control	Case control	Case control
Disease type	AD	AD	AD	MCI	AD+ aMCI
Participants	AD + HC	AD	AD + HC	MCI + HC	AD + aMCI + HC
Experimental group (n)	10 Male: 6 (60.0%) Female: 4 (40.0%)	16 Male: 7 (43.8%) Female: 9 (56.3%)	15 Male: 8 (53.3%) Female: 7 (46.7%)	24 Male: 11 (45.8%) Female: 13 (54.2%)	30 aMCI: Male: 4 (26.7%) Female: 11 (73.3%) AD: Male: 5 (33.3%) Female: 10 (66.7%)
Experimental group mean age	79.7 ± 5.1	76.5 ± 6.8	69.5 ± 6.8	73.7 ± 1.0	aMCI: 69.4 ± 5.8 AD: 72.8 ± 6.1
Control group (n)	14 Male: 9 (64.3%) Female: 5 (35.7%)	16 Male: 9 (56.3%) Female: 7 (43.8%)	10 Male: 5 (50.0%) Female: 5 (50.0%)	24 Male: 8 (33.3%) Female: 16 (66.7%)	23 Male: 8 (34.8%) Female: 15 (65.2%)
Control group mean age	77.0 ± 7.1	76.4 ± 6.0	71.1 ± 5.9	69.0 ± 1.1	67.1 ± 6.9
Overall mean age	78.1	76.4	70.14	71.35	69.37

**Table 2:** PAS interventions in the included studies.

Intervention characteristics	Terranova et al. (2013)	Kumar et al. (2023)	Di Lorenzo et al. (2018)	Lahr et al. (2016)	Meder et al. (2021)
Intervention method	rPAS	rPAS	cc-PAS	PAS	PAS LTP
Target area	M1	DLPFC	PPC-M1	M1	M1
Stimulation paradigm	Paired TMS + PNS	Paired TMS + PNS	Paired TMS-TMS	Paired TMS + PNS	Paired TMS + PNS
Key ISI	25 ms (PNS before TMS)	Active:25 ms; Control:100 ms	±5 ms (PPC-M1 order variant)	25 ms (PNS before TMS)	25 ms (PNS before TMS)
Stimulation frequency	5 Hz	0.1 Hz	0.2 Hz	0.1 Hz	0.25 Hz
Pulses per session	600 pairs	180 pairs	100 pairs	180 pairs	225 pairs
Total sessions	Single session	10 sessions (2 weeks)	Single session	Single session	Single session (2 visits)
TMS intensity	90% AMT	Intensity to evoke 1 mV MEP	PPC: 90% RMT; M1: 1 mV MEP intensity	Intensity to evoke 1 mV MEP	SI1mV intensity
Electrical stimulation intensity	200% sensory threshold	300% sensory threshold	No electrical stimulation	300% Sensory Threshold	300% Sensory Threshold
Attention control	No attention control task	counting sensory stimuli	No attention control task	Visual counting task (blue balls)	counting number of stimuli
Safety	Safe, no serious adverse events	Safe, no serious adverse events	Safe, no serious adverse events	Safe, no serious adverse events	Safe, no serious adverse events

**Table 3:** Outcome measures.

Outcome measures	Terranova et al. (2013)	Kumar et al. (2023)	Di Lorenzo et al. (2018)	Lahr et al. (2016)	Meder et al. (2021)
Primary outcome	MEP amplitude change, SAI change	DLPFC plasticity; Working Memory	MEP amplitude change (spike-timing dependent plasticity (STDP))	MEP amplitude change (PAS response rate)	MEP amplitude change (LTP-like plasticity)
Secondary outcome	RMT change	Theta-gamma coupling	N/A	Hippocampal volume, genotype, etc.	Cortical excitability, CSF markers, etc.
Primary outcome change	AD patients did not show rPAS-induced plasticity	Active rPAS transiently enhanced DLPFC plasticity, working memory & oscillatory coupling	Impaired STDP-like plasticity in AD patients’ PPC-M1 pathway	No significant PAS effect difference between MCI and HC groups	No plasticity difference among groups, but cortical spinal hyperexcitability in aMCI
Secondary outcome change	AD group RMT significantly lower than HC	Neural oscillatory coupling positively correlated with working memory performance	N/A	Within MCI group, PAS effect correlated with subjective alertness & sleep duration	PAS effect not significantly correlated with CSF biomarkers or cognition

Heterogeneity among MEP-based studies was statistically low (*Chi^2^ =* 3.23*, df =* 3*, p =* 0.36*, I^2^ =* 7%), but this should not be interpreted as evidence of physiological equivalence across PAS paradigms. Given the small number of studies and the differences in protocols, disease stage, and outcome definitions, the pooled effect was interpreted cautiously as an exploratory estimate.

### 3.3 Risk of bias assessment

**Figure 2:**
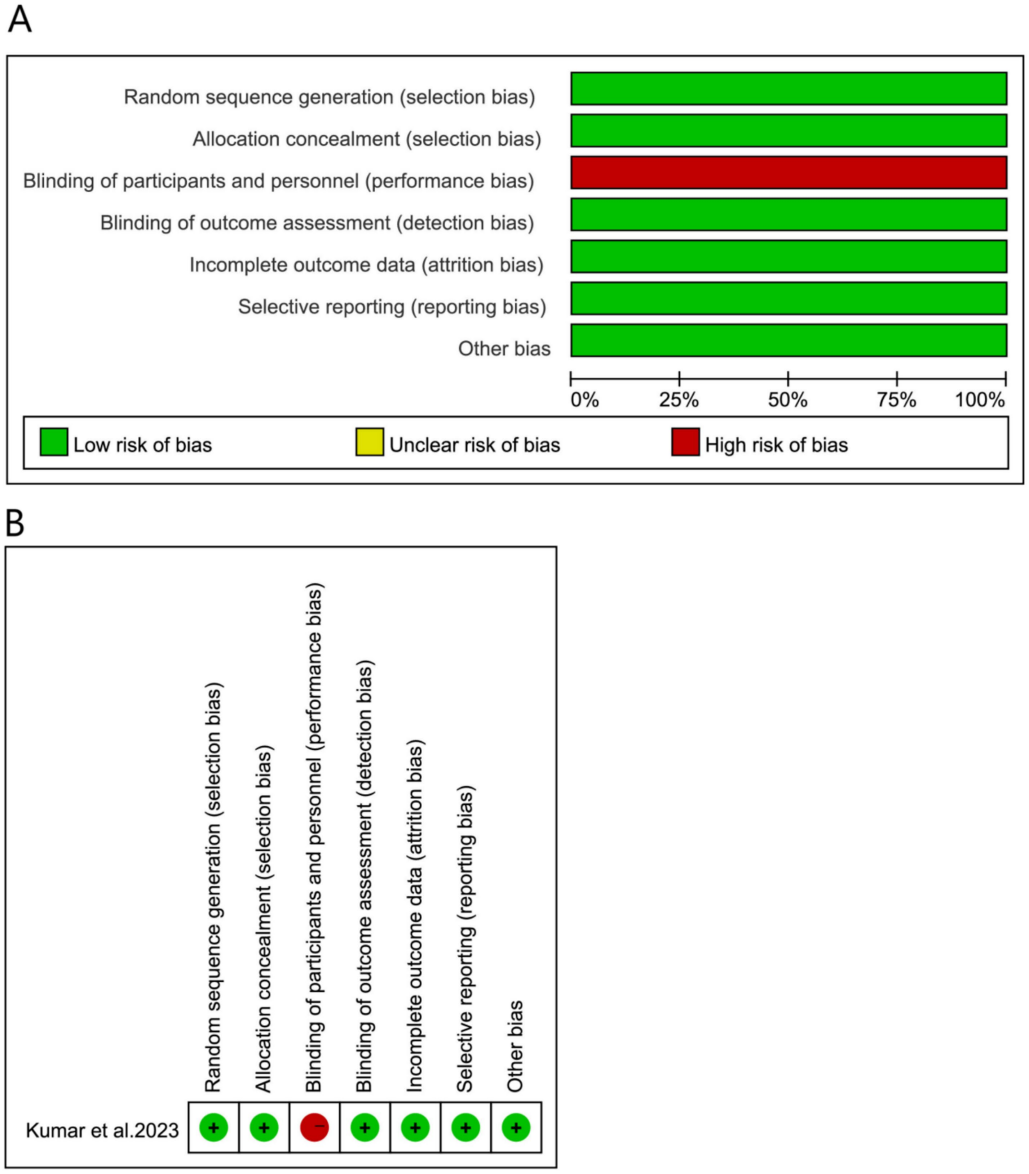
Risk of bias 2 for Kumar et al. (2023). **(A)** Summary of risk of bias judgments across domains. **(B)** Risk of bias judgments for the individual study across domains.

Among the four case–control studies, two were rated as excellent quality (NOS = 8 or 9 out of 9), and two as good quality (NOS = 7); these assessments are summarized in Table 4, indicating relatively high methodological quality. The single randomized controlled trial, assessed with Risk of Bias 2 (RoB 2), showed a low overall risk of bias (Figure 2). In summary, most included studies demonstrated a low risk of bias in our quantitative analysis.

**Table 4:** Newcastle Ottawa-Scale for case–control studies.

Study	Selection				Comparability	Outcome			Scores
Terranova et al. (2013)	☆	☆	☆	☆	☆☆	☆	☆	☆	9
Lahr et al. (2016)	☆	☆	☆	☆	☆	☆	☆		7
Meder et al. (2021)	☆	☆		☆	☆☆	☆	☆		7
Di Lorenzo et al. (2018)	☆	☆	☆	☆	☆☆	☆	☆		8

### 3.4 Adverse events

All five studies included monitoring of safety during PAS interventions. Meder et al. explicitly reported that all participants tolerated PAS well, with no intervention-related adverse events. Similarly, Terranova et al. (2013) noted that all subjects completed the entire PAS session without any significant discomfort or adverse effects. Lahr et al. (2016) and Di Lorenzo et al. (2018) did not separately list safety outcomes but reported no adverse events during the experimental procedures, and all subjects completed the protocol as planned. Kumar et al. (2023) did report some minor transient adverse events: mild headaches (2 cases in the treatment group and 1 in the control), mild pain or discomfort (3 or 4 cases), and fatigue (1 case in each group). However, all these reactions were brief and mild, and no serious adverse events or early withdrawals occurred. Overall, PAS was well tolerated in all included studies.

### 3.5 Quantitative and descriptive analysis results

**Figure 3:**
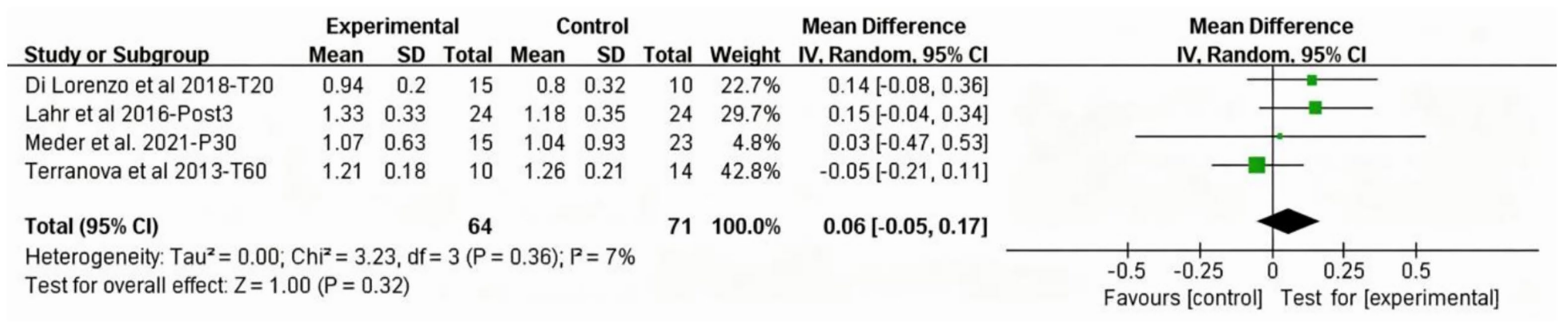
Forest plot of the exploratory meta-analysis of MEP-based PAS outcomes in people with MCI or AD and healthy controls. Mean difference estimates and corresponding 95% confidence intervals are shown. This analysis was restricted to studies reporting extractable MEP amplitude changes after PAS and should be interpreted in the context of the limited number of studies and methodological heterogeneity.

To quantitatively assess the overall effect of PAS on cortical plasticity in people with MCI or AD, we conducted an exploratory random-effects meta-analysis of the four case–control studies reporting extractable changes in MEP amplitude after PAS. We used the change in MEP amplitude at a specific post-intervention time point selected by each study (15–60 min after PAS) relative to baseline as the outcome measure. Data were available for 64 patients with MCI or AD and 71 healthy controls. The pooled result showed no statistically significant difference in PAS-induced MEP changes between patients and controls [MD = 0.06, 95% CI (−0.05, 0.17), *p* = 0.32], as illustrated in Figure 3. This negative finding should not be interpreted as evidence of preserved cortical plasticity in MCI or AD; rather, it reflects insufficient MEP-based evidence for a consistent group difference.

In these studies, cortical plasticity was assessed using PAS protocols in people with MCI or AD. Di Lorenzo et al. (2018) utilized the cc-PAS technique to investigate intercortical plasticity in patients with AD, with MEP amplitude as the core indicator. Healthy older adults exhibited bidirectional modulation of MEP amplitude consistent with STDP, whereas the same stimulation protocol failed to induce significant MEP changes in AD patients. Terranova et al. (2013) assessed sensory-motor plasticity in patients with mild AD through high-frequency rPAS. Their study demonstrated that healthy elderly subjects exhibited an increase in MEP amplitude after stimulation, whereas patients with mild AD showed no such change in MEP amplitude. Lahr et al. (2016) compared cortical plasticity between patients with MCI and healthy elderly individuals using the traditional PAS paradigm. They found no significant differences in the amplitude changes of MEPs between the two groups after stimulation, and the changes in MEPs amplitude were not correlated with multiple clinical markers of disease severity. The latest study by Meder et al. (2021) further systematically evaluated the reliability of this paradigm. They found that standard PAS failed to consistently enhance MEPs in patients, with high individual variability and low test–retest reliability. Separately, using stimulus intensity metrics, the study identified corticospinal hyperexcitability. These findings indicate that PAS results vary by protocol, circuit, and disease stage.

### 3.6 Narrative synthesis of the single randomized controlled trial

In the single included RCT (Kumar et al., 2023), rPAS targeting the DLPFC plasticity did not show a significant group-by-time effect on prefrontal plasticity, working memory, or theta-gamma coupling compared with control stimulation. Exploratory within-group findings suggested transient modulation of DLPFC plasticity and working memory after active rPAS, but these effects were not sustained. Therefore, this study provides preliminary, hypothesis-generating evidence that prefrontal rPAS may acutely engage cognition-related networks, but the tested protocol did not demonstrate durable superiority over control stimulation.

## 4 Discussion

The meta-analysis showed no significant overall effect in MEP-measured PAS responses, but this finding should be interpreted cautiously. The included studies differed in PAS paradigm, cortical target, disease stage, and theoretical assumptions regarding plasticity. Therefore, the pooled estimate should be understood as an exploratory synthesis of motor-system MEP readouts rather than as evidence of preserved global cortical plasticity in MCI or AD. The divergent findings across individual studies likely reflect methodological heterogeneity, biological heterogeneity, and circuit-specific vulnerability across the AD continuum.

### 4.1 Circuit specificity and limitations of MEP-based motor-system readouts

The assessment of cortical plasticity exhibits high regional and circuit specificity. Classical peripheral nerve-M1 PAS, cc-PAS targeting PPC-M1 pathways, and prefrontal rPAS paradigms engage partially different sensorimotor, cortico-cortical, and higher-order associative networks (Guidali et al., 2021a,b). Accordingly, MEP modulation should not be treated as a direct or comprehensive proxy for cortical synaptic plasticity. MEP primarily reflects plasticity-related changes within the primary motor cortex, corticospinal pathways, and motor output system (Bestmann and Krakauer, 2015). Recent TMS-EEG evidence further suggests that cortical and corticospinal readouts may provide complementary but not interchangeable information about PAS-induced plasticity (Arrigoni et al., 2026; Conde et al., 2019).

This distinction is particularly important in MCI and AD, because early AD pathology preferentially affects medial temporal and associative cortical networks, whereas primary motor cortex involvement may be less prominent in early disease stages (Schöll et al., 2016). Therefore, preserved or non-significantly altered MEP responses cannot exclude plasticity abnormalities in cognition-related circuits. The absence of a group difference in Lahr et al. (2016) may reflect relatively preserved M1-related plasticity at the MCI stage, but may also indicate limited sensitivity of MEP-based PAS to subtle non-motor network dysfunction. In contrast, Di Lorenzo et al. (2018) showed impaired STDP-like plasticity in the PPC-M1 pathway, a parieto-motor circuit previously reported to be altered in AD (Bonnì et al., 2013), whereas Terranova et al. (2013) reported absent rPAS-induced sensorimotor plasticity in mild AD.

Taken together, these findings support a circuit-specific interpretation. MEP-based PAS remains useful for probing motor-system plasticity, but it provides only a partial and regionally constrained index of plasticity-related processes in cognitive neurodegeneration. Therefore, pooled MEP-based findings should not be interpreted as definitive evidence for preserved or impaired cortical plasticity across the AD continuum.

### 4.2 Heterogeneity of PAS protocol parameters: methodological differences and challenges in data integration

PAS-induced plasticity effects are highly dependent on stimulation parameters, and the heterogeneity of existing protocols directly affects the comparability and interpretation of findings (Wolters et al., 2003; Müller-Dahlhaus and Ziemann, 2015). The studies included in this review differed substantially in key parameters. Di Lorenzo et al. (2018) employed cc-PAS targeting the PPC-M1 circuit with interstimulus intervals (ISIs) of ±5 ms; Lahr et al. (2016) used the classical peripheral nerve-M1 PAS with an ISI of 25 ms; Terranova et al. (2013) applied high-frequency rPAS at 5 Hz; whereas Kumar et al. (2023) implemented low-frequency rPAS at 0.1 Hz with a multi-day intervention paradigm. Variations in ISI, stimulation frequency, stimulation target, and intervention mode are likely to engage partly distinct plasticity mechanisms, including protocol- and region-dependent neural responses. Therefore, the results of these studies should not be interpreted as interchangeable measures of a single PAS-induced plasticity construct (Ridding and Ziemann, 2010; Ong and Tang, 2025).

Importantly, baseline cortical excitability in patients with AD may already be altered. Meder et al. (2021) reported that, although no significant differences in PAS-induced MEP changes were observed among aMCI, AD, and healthy control groups, altered corticospinal excitability was already present at the aMCI stage. This finding suggests that standard PAS parameters optimized in healthy individuals may not be well matched to the altered neurophysiological state of patients along the MCI to AD continuum, potentially resulting in attenuated, absent, or variable plasticity induction (Ridding and Ziemann, 2010; Hamada et al., 2013). In addition, pre-existing interindividual differences in structural connectivity within neural circuits can substantially influence PAS responsiveness, further increasing variability across studies (Jack et al., 2010).

### 4.3 Disease-stage and biological heterogeneity

Clinical diagnoses of MCI, aMCI, and AD may not correspond to biologically homogeneous disease entities. Across the included studies, biomarker confirmation of AD pathology using CSF, PET, or other AD-specific markers was inconsistently available, which limits the interpretation of disease-specific PAS alterations. Participants with similar clinical labels may differ in amyloid and tau status, vascular burden, cognitive reserve, medication exposure, disease stage, and the degree of network involvement (Jack et al., 2010; Braak and Braak, 1991; Bondi and Smith, 2014; Petersen et al., 2009). Such biological heterogeneity can dilute group-level effects and partly explain the divergent PAS findings across studies.

PAS abnormalities may therefore emerge preferentially in specific biological subtypes, disease stages, or network phenotypes, rather than representing a uniform feature of clinically defined MCI or AD. For example, Lahr et al. (2016) reported no significant MEP-based plasticity difference in MCI, whereas Di Lorenzo et al. (2018) and Terranova et al. (2013) reported impaired responses in cortico-cortical or sensorimotor circuits in mild AD. Meder et al. (2021), which included both aMCI and AD participants, reported overall negative PAS findings but showed altered baseline corticospinal excitability. These observations suggest that excitability and plasticity abnormalities may not evolve linearly across clinical categories.

Taken together, the current meta-analysis does not provide sufficient evidence to establish a stage-dependent decline in PAS-induced motor-system plasticity. Because biomarker-confirmed AD pathology was not consistently available, the pooled estimate should be interpreted as an exploratory synthesis of clinically defined people with MCI or AD cohorts rather than a definitive estimate of PAS abnormalities in biologically confirmed AD. Future longitudinal studies should recruit biomarker-characterized cohorts, stratify participants by amyloid or tau status, disease stage, and network phenotype, and combine PAS with multimodal neurophysiological and neuroimaging assessments, such as TMS-EEG, fMRI, and diffusion imaging, to clarify how circuit-specific plasticity evolves across disease progression (Arrigoni et al., 2026; Ferreri et al., 2021; Tăuƫan et al., 2023).

### 4.4 Compensatory, maladaptive, and state-dependent interpretations of PAS responses

PAS responses in MCI and AD should not be interpreted within a simple dichotomy of preserved versus impaired plasticity. Preserved or enhanced MEP facilitation may reflect compensatory recruitment, reduced inhibitory control, maladaptive cortical hyperexcitability, or altered homeostatic/metaplastic regulation rather than intact physiological plasticity. Conversely, absent or attenuated PAS responses may result not only from impaired synaptic plasticity, but also from altered baseline excitability, homeostatic saturation, or insufficient engagement of the targeted circuit (Müller-Dahlhaus and Ziemann, 2015; Hamada et al., 2013).

This framework is particularly relevant to early AD, where synaptic dysfunction may coexist with excitation-inhibition imbalance, cholinergic dysregulation, corticospinal hyperexcitability, and large-scale network instability (Hoover et al., 2010; Koch et al., 2011; Lawson et al., 2025). Under such conditions, PAS responsiveness may be strongly state-dependent: the direction, magnitude, and reliability of PAS-induced after-effects may vary according to baseline excitability, oscillatory activity, attention, aging-related plasticity changes, structural connectivity, disease stage, and network vulnerability (Ridding and Ziemann, 2010; Di Fazio et al., 2026; Freitas et al., 2011b; Fathi et al., 2010). Therefore, apparently preserved or exaggerated PAS responses should not be equated with preserved physiological plasticity, and reduced responses should not be interpreted without considering the baseline neurophysiological state.

Viewing PAS through this broader framework may help reconcile divergent findings across studies. It suggests that PAS abnormalities in MCI and AD may reflect not only synaptic plasticity impairment, but also maladaptive hyperexcitability and altered metaplastic regulation within vulnerable networks. Future studies should combine PAS with TMS-EEG, neuroimaging, and molecular biomarkers to differentiate compensatory plasticity, maladaptive excitability, and true synaptic plasticity impairment (Tăuƫan et al., 2023).

### 4.5 Limitations of the study

Several limitations should be considered when interpreting the findings of this review. First, the evidence base was limited, with only five included studies and modest sample sizes. This reduced statistical power and increased uncertainty around the pooled estimate. Therefore, the non-significant meta-analytic finding should be interpreted as exploratory rather than as definitive evidence for the absence of PAS-induced plasticity alterations in MCI or AD. Second, substantial methodological heterogeneity was present across the included studies. The studies differed in PAS paradigm, interstimulus interval, stimulation frequency, cortical target, number of sessions, and outcome definition. Classical peripheral nerve-M1 PAS, cc-PAS, and prefrontal rPAS likely probe partially different neural circuits and plasticity mechanisms, which limits direct comparability and constrains the interpretation of the pooled MEP-based analysis (Wolters et al., 2003; Müller-Dahlhaus and Ziemann, 2015; Ridding and Ziemann, 2010). Third, disease-related and biological heterogeneity may have affected the findings. Biomarker confirmation of AD pathology was not consistently available, and clinically defined MCI or AD cohorts may have differed in amyloid/tau status, disease stage, vascular burden, cognitive reserve, medication exposure, and network involvement (Jack et al., 2018; Koch et al., 2011). Fourth, there is a general lack of long-term longitudinal data. Apart from the two-week intervention study conducted by Kumar et al. (2023), the remaining studies all employed single-case control designs, which cannot depict the dynamic evolution of cortical plasticity during the disease process, nor can they assess its predictive value for clinical outcomes. Finally, potential confounding factors such as medication use, vascular risk factors, depressive symptoms, sleep quality, attention, and baseline neurophysiological state were not consistently reported or controlled. These factors may have contributed to interindividual variability and limited the disease-specific interpretation of PAS responses. The studies exhibited inadequate control over potential confounding factors (Fathi et al., 2010; Inghilleri et al., 2004).

### 4.6 Future outlook and challenges

Future studies should address several methodological and conceptual challenges. First, PAS research in MCI and AD should move beyond single MEP-based outcomes and incorporate multimodal approaches (Freitas et al., 2011a), such as TMS-EEG, neuroimaging, and AD-related molecular biomarkers, to better distinguish motor-system excitability changes from cortical and network-level plasticity alterations (Arrigoni et al., 2026; Tăuƫan et al., 2023; Di Fazio et al., 2026). Second, future work should consider the circuit-specific and state-dependent nature of PAS responses. Classical peripheral nerve-M1 PAS, cc-PAS, and prefrontal rPAS likely probe different neural circuits and should not be interpreted as interchangeable measures of a single plasticity construct (Guidali et al., 2021a; Di Lorenzo et al., 2018). Baseline excitability, oscillatory state, attention, sleep, medication use, and structural connectivity may all influence PAS responsiveness and should be systematically reported or controlled (Müller-Dahlhaus and Ziemann, 2015; Ridding and Ziemann, 2010; Di Fazio et al., 2026). Finally, larger longitudinal and biomarker-characterized studies are needed to determine whether PAS abnormalities are associated with AD-specific pathology, disease progression, or treatment response. Although preliminary rPAS evidence suggests possible modulation of prefrontal plasticity and cognition, adequately powered trials with optimized protocols are required before clinical efficacy can be inferred (Kumar et al., 2023; Di Fazio and Palermo, 2026).

## 5 Conclusion

Current MEP-based evidence is insufficient to establish a consistent difference in PAS-induced motor-system plasticity between individuals with MCI/AD and healthy older adults. This finding should not be interpreted as evidence of preserved cortical plasticity, given the small number of studies, protocol heterogeneity, circuit-specific differences among PAS paradigms, and the limited interpretability of MEP-based outcomes. Corticospinal hyperexcitability and network-level PAS findings may represent candidate neurophysiological observations, but they remain preliminary and require validation in larger, longitudinal, and biomarker-characterized cohorts.

## Data Availability

The original contributions presented in the study are included in the article/supplementary material, further inquiries can be directed to the corresponding authors.
